# Parenting an Adolescent: The Case of the Avoidant Highly Sensitive Mother

**DOI:** 10.1007/s10578-024-01761-8

**Published:** 2024-09-20

**Authors:** Alon Goldberg, Alexander Zibenberg

**Affiliations:** 1 Department of Education, Tel-Hai College, Upper Galilee, Qiryat Shemona, 12210 Israel; 2 Department of Education, Department of Human Resources, Tel-Hai College, Upper, Qiryat Shemona, Galilee 12210 Israel

**Keywords:** Highly sensitive person, Attachment avoidance, Adolescence, Parenting practices

## Abstract

This research examines parental practices of Israeli highly sensitive mothers toward their adolescent children and the role of attachment avoidance as a moderator between the associations of high sensitivity and parenting practices. One hundred and one mother–adolescent dyads completed self-report questionnaires assessing mothers’ degree of high sensitivity, mothers’ adult attachment, and mothers’ parenting practices. Results showed that highly sensitive mothers were described by their adolescent children as inconsistent and intrusive parents. Further, attachment avoidance was found to moderate the association between mothers’ high sensitivity and inconsistent and psychological intrusiveness. Findings suggest that attachment avoidant highly sensitive mothers experience this period of raising adolescents as especially stressful and challenging, which contributes to the practice of negative parenting. Thus, interventions focused on regulating those mothers’ emotions to better cope with parental challenges could buffer negative parenting practices.

In Western societies, adolescence is an especially arduous period for adolescents and parents because of major biological, cognitive, and emotional changes the adolescents are undergoing; changes in parent–adolescent relationships [1]; and adolescents’ growing need for autonomy, individuation, and more egalitarian relationships [2] that might increase parent–adolescent conflict [3]. Also, adolescents spend less time with their parents and more time with peers [4], making it more challenging for parents to monitor their adolescents’ whereabouts. Furthermore, adolescence usually overlaps with parents’ midlife needs, when they are reevaluating their life course [5] and going through what has been found to be the lowest point in parents life satisfaction [6]. Based on the determinants of the parenting model proposed by Belsky [7, 8], parental personality and parental psychological functioning are the most significant factors in the determinants of parenting, because they affect parenting behavior and predict optimal parental behaviors [7, 8]. Hence, the current study seeks to add to the literature examining parenting behaviors (i.e., parenting practices) during the challenging time of parenting adolescents, by analyzing the characteristics and parenting practices of highly sensitive mothers with attachment avoidant orientation, which has not yet been explored.

This research was conducted in Israel with Israeli participants. Israeli society is an intermediate one somewhere along the individualism-collectivism axis with strong family values and connectedness. As studies have reported, Israel has high levels of closeness between parents and children and autonomy support, but its highly involved parents have difficulties in practicing parental authority and parental monitoring [9, 10].

Being highly sensitive refers to an individual’s temperamental trait, genetically based, to tend to process more strongly and deeply a variety of information, such as one’s own and others’ moods and thoughts. Highly Sensitive Persons (HSPs), who constitute 20-30% of the population [11], tend to process and respond to lower thresholds of information and to better detect subtle differences in their environment [12, 13]. HSPs are more likely to experience social phobia [14], depression [15], avoidant personality disorder [16], behavioral inhibition [17], and attachment insecurity [18]. However, HSPs are not condemned to have “challenging” personalities with associated negative effects. There is some evidence that HSPs are differentially susceptible to environmental factors [19, 20]. Studies on highly sensitive children show that their sensitivity magnifies both positive and negative effects of their early relationships with their significant others [21, 22] as they integrate those experiences into their personality, coping strategies, and behaviors [19, 20, 23–25]. Thus, it is important to explore highly sensitive parents’ adjustment during the challenging period of adolescence.

In the past several years, there have been some research findings regarding the characteristics of highly sensitive parents, most of them based on mothers’ self-reports. An exploratory study that examined the subjective experience of highly sensitive parents found that they had more attunement with their young children, although mothers found parenthood more difficult [26]. In a qualitative study exploring the parenting experiences of highly sensitive mothers during the postnatal period, highly sensitive mothers reported heightened awareness, attunement, and responsiveness to their infants’ needs. However, they acknowledged the challenges of their sensitivity affecting their ability to navigate motherhood [27]. An examination of the parenting style of highly sensitive parents suggested that the parenting challenges may drive the highly sensitive parent to either a permissive or authoritarian parenting style, both of which are not optimal parenting styles [28]. These findings are consistent with a prior study that found that some highly sensitive mothers were emotionally overwhelmed and adopted harsh parenting behavior, spending time alone, withdrawing from the child, giving less focus and attention to the child’s needs, and showing more permissive parenting behavior [29], which might be experienced by the child as inconsistent parenting.

## Attachment, Parenting, and HSPs

Attachment relationships function as an inner resource when coping with stressful events, leading people to maintain or restore proximity to significant others who can provide support and comfort when one is distressed [30]. According to Bowlby [30], attachment relationships are “cradle to grave” relationships, which are shaped by the end of infancy and tend to be stable over one’s life span. During infancy, proximity maintenance with attachment figures may relieve distress and facilitate positive representations about the self and others; further encourage individuals to turn to others when distressed, using their support effectively; and internalize a “secure attachment” representation [31]. However, when significant others are unavailable or nonresponsive in times of need, distress increases, encouraging the formation of insecure attachment [31].

Two dimensions of attachment that reflect insecurity are anxiety and avoidance. Anxious attachment individuals tend to be more self-centered, worry about their own attachment needs, feel extremely distressed when other people need their assistance, and adopt a hyperactivating strategy of emotion regulation [32, 33], while avoidant attachment individuals tend to feel discomfort with emotional closeness and dependence on intimate partners and use a deactivating strategy of emotion regulation.

The literature suggests that being a highly sensitive child probably plays a role in the way that HSPs processed their early experiences with their significant others, likely feeling misunderstood by their significant others and thus receiving poor attunement to their needs [29]. Hence, highly sensitive children might develop attachment insecurity through their adulthood. However, findings regarding adult HSPs and attachment insecurity are inconsistent, as research shows that high sensitivity consistently associates with attachment anxiety (e.g [28]). while attachment avoidance associates with high sensitivity using only Dunn`s model [34] and the Adolescent/Adult Sensory Profile (AASP; [35]), but not using the Aron and Aron conceptualization [36] and the Highly Sensitive Person Scale [18].

One investigation focused on parents of adolescents and examined the parenting of highly sensitive parents and their attachment, finding parents being highly sensitive was positively associated with attachment anxiety, and attachment anxiety mediated the association between parents’ high sensitivity and harsh parenting and partially mediated the association between parents’ high sensitivity and parental psychological intrusiveness [37]. The current study expands on those findings by exploring the effect of attachment avoidance among those highly sensitive parents and by analyzing only the mother–adolescent dyads. The lack of consistent association in the literature between attachment avoidance (as opposed to attachment anxiety) and high sensitivity encouraged us to reexamine the data in that previous study to explore the avoidant tendency of highly sensitive parents. We focused on the mother–adolescent dyads to allow better comparison with studies in the field, which mostly study mothers.

There is substantial empirical evidence that attachment avoidance influences adult functioning such as parenting, which requires typical information processing, emotion regulation, and coping strategies [32, 38, 39]. Parental avoidant attachment orientation may prompt the parent to view the child’s temperamental needs as a threat and misinterpret them and thus limit their involvement to avoid the distress caused by failed efforts to cope [40]. Hence, the avoidant parents, who use deactivation emotion regulation strategies, may believe that the best way to maintain their safety is by not depending on others or not allowing others to depend on them, which causes their parenting to be less caring and responsive [41], inconsistent [42], and more harsh [43].

## The Current Study

The current study examines whether attachment avoidance plays a part in shaping parental practices of highly sensitive mothers due to highly sensitive persons’ differential susceptibility to environmental factors [20], magnifying the negative effects of avoidant attachment experiences and integrating them into their coping strategies and behavior [19, 20, 24, 25]. It is possible that where attachment avoidance is high, the association between high sensitivity and negative parental practices (inconsistency, harsh discipline, and psychological intrusiveness) may be strengthened. However, when attachment avoidance is low, the association between high sensitivity and negative parental practices may be weakened. Therefore, we hypothesized the following:

### H1

A mother being an HSP will be positively associated with inconsistent, harsh, and intrusive parenting.

### H2

Avoidant attachment will moderate the relationship between an HSP and negative parenting practices (inconsistency, harsh discipline, and psychological intrusiveness), so that the relationship will be stronger for individuals with high levels of avoidance.

**Fig. 1:**
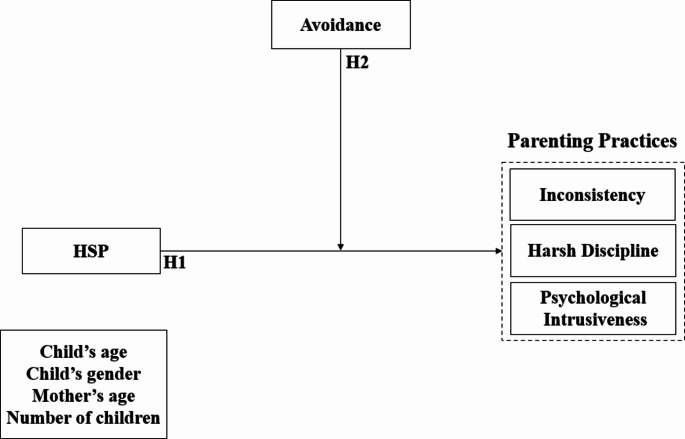
Research model

Fig. 1 displays the components of our research model.

## Method

### Participants

Participants were 101 Israeli mother–adolescent dyads. Of the participants, 97% were Jewish and 3% were Muslim or Druze. The mean age of the mothers was 43.4 years (*SD* = 5.59) and they had an average of 2.85 children (*SD* = 1.04). Of the mothers, 82% were married and 18% were single parents, 80.1% were secular, 88.1% had a graduate/professional degree, and 94% reported an average or above average income (average monthly income in Israel is approximately $3430; [44]. Adolescent participants were 35.6% male and 64.4% female, with a mean age of 13.91 years (*SD* = 1.58); 53.1% were the eldest child in their family, and 27.6% were the youngest child.

### Procedure

Israeli mothers of adolescents between the age of 12 and 18 were invited through social media to participate in an online study presented as “parenting characteristics and parenting adolescents.” After signing digital informed consent forms, adolescents and mothers received separate links and were asked to complete online questionnaires separately. Mothers completed a demographic questionnaire, the short version of the Highly Sensitive Person Scale (HSP-12), and the Experiences in Close Relationships Scale (ECRS). Adolescents completed the Weinberger Parenting Inventory (WPI) regarding their mother. Participants were informed that their anonymity would be preserved throughout the study and that they had the right to discontinue participation at any time. There were 18 additional mother–adolescent dyads who initially signed consent forms, but they filled out only a few items (less than 20) on the questionnaires and therefore were dropped from the study, there not being enough data from them to compare with those who completed the questionnaires/reports.

### Instruments

#### Demographic Questionnaire

We collected information about the adolescents’ gender (female\male) and age; mothers’ religion, marital status, age, and education; the family’s income level; and the number of children in the family.

#### Highly Sensitive Person Scale - HSP-12

High sensitivity was measured with the Hebrew version of the self-report HSP 12-item scale [20], which is based on the original 27-item scale (the HSPS; [36]), which has been highly validated (e.g [45–47]). Participants were asked to respond using a Likert scale ranging from 1 (*not at all*) to 7 (*extremely*) (e.g., “Are you annoyed when people try to get you to do too many things at once?”). In the present study, the Cronbach’s alpha was 0.90. Items were averaged to obtain a total mean score.

#### Experiences in Close Relationships Scale (ECRS)

The ECRS [48] is a 36-item self-report questionnaire assessing attachment-related avoidance (e.g., “I prefer not to show a partner how I feel deep down”) and anxiety (e.g., “I worry about being abandoned”), rated on a Likert scale ranging from 1 (*not at all*) to 7 (*very much*,* strongly agree*). We used the Hebrew version, which was found to be highly reliable and valid [49]. In the present study, the Cronbach’s alpha for the avoidance scale was 0.90 and for the anxiety scale was 0.91. We used only the avoidance scale. A score of 4 on the scale was used as a cut-off regarding high and low attachment avoidance.

### Weinberger Parenting Inventory (WPI)

The WPI [50] is a 50-item adolescent-report instrument that assesses adolescents’ perception of how they are being parented. It assesses child-centeredness, permissiveness, psychological intrusiveness, harsh discipline, and inconsistency. In the current study, adolescents reported the parenting practices of their mothers that they perceived and assessed their mothers’ psychological intrusiveness (e.g., “she tries to manage my life more than she should”; *α* = 0.87), harsh discipline (e.g., “I feel the punishment she gives me is unfair”; *α* = 0.71), and inconsistency (e.g., “she tells me one thing and does another”; *α* = 0.78). We used the Hebrew version, which has been found to be valid and reliable (e.g [47, 51]).

### Data Analysis

To test the study hypotheses, we used the SPSS software package, which included the PROCESS package. For the first hypothesis, in predicting three parenting practices by HSP, we controlled for the child’s age and gender, the mother’s age, and the number of children, as we assumed these factors might influence the dependent variables. We ran three regressions, each for separate parenting practices. For the second hypothesis, we followed Aiken et al. [52] guidelines using the PROCESS package for moderation analysis. We performed a moderation analysis using SPSS Macro PROCESS [53]. In this analysis, HSP was an independent variable (X), avoidance was a moderator (M), and inconsistency with psychological intrusiveness were treated as dependent variables (Y).

## Results

As shown in Table 1, HSP positively correlated with two practices (inconsistency and psychological intrusiveness). These correlations provide preliminary support for H1. However, there was no significant effect between HSP and the practice of harsh discipline. Means, standard deviations, and correlations among study variables are presented in Table 1.

**Table 1:** Means, standard deviations, and correlations among variables

Variables	M	SD	1	2	3	4	5	6	7	8
1. Inconsistency	2.32	0.68								
2. Psychological intrusiveness	2.64	0.49	0.56**							
3. Harsh discipline	1.60	0.58	0.53**	0.37**						
4. HSP	4.68	0.90	0.28**	0.37**	− 0.01					
5. Avoidance (1-high; 0-low)	0.49	0.50	0.13	0.04	− 0.05	0.34**				
6. Child’s age	13.91	2.59	− 0.12	− 0.01	0.08	− 0.20*	− 0.16			
7. Child’s gender (1-male)	0.36	0.48	− 0.07	− 0.06	− 0.05	− 0.04	− 0.10	− 0.05		
8. Mother’s age	43.54	5.59	− 0.05	− 0.04	− 0.05	− 0.12	− 0.07	0.29**	0.02	
9. Number of children	2.85	1.04	− 0.15	− 0.03	− 0.08	− 0.21*	− 0.02	0.25**	− 0.03	0.09

*p < .05; **p < .01

We used the SPSS software package to test the first hypothesis concerning the impact of HSP on inconsistency and psychological intrusiveness, while controlling for age of child, child’s gender, mother’s age and number of children. The regression coefficients are presented in Table 2. Positive effect was found for HSP with inconsistency (*β* = 0.19, *p* < .05) and psychological intrusiveness (*β* = 0.21, *p* <. 01). As anticipated from the correlation results (see Table 1), no significant effect of HSP on harsh discipline was observed. H1 was partly supported.

**Table 2:** Estimation of inconsistency with psychological intrusiveness

Variables	Inconsistency β (SE)	Intrusivenessβ (SE)	Harsh disciplineβ (SE)
HSP	0.19* (0.08)	0.21** (0.05)	− 0.01 (0.07)
Child’s age	− 0.02 (0.03)	0.02 (0.02)	0.03 (0.03)
Child’s gender (1-male)	− 0.08 (0.14)	− 0.04 (0.10)	0.06 (0.12)
Mother’s age	0.01 (0.01)	− 0.01 (0.01)	− 0.01 (0.01)
Number of children	− 0.06 (0.07)	0.02 (0.05)	− 0.06 (0.06)

*p < .05; **p < .01

To test H2, we conducted Aiken et al. [52] procedure for testing interaction effects. We performed a moderation analysis using SPSS Macro PROCESS [53]. In this analysis, HSP was an independent variable (X), avoidance was a moderator (M), while inconsistency with psychological intrusiveness were treated as dependent variables (Y). Since there was no direct effect of HSP on harsh discipline, we did not pursue that interaction effect. To interpret these significant interactions, we plotted the relationships.

**Fig. 2:**
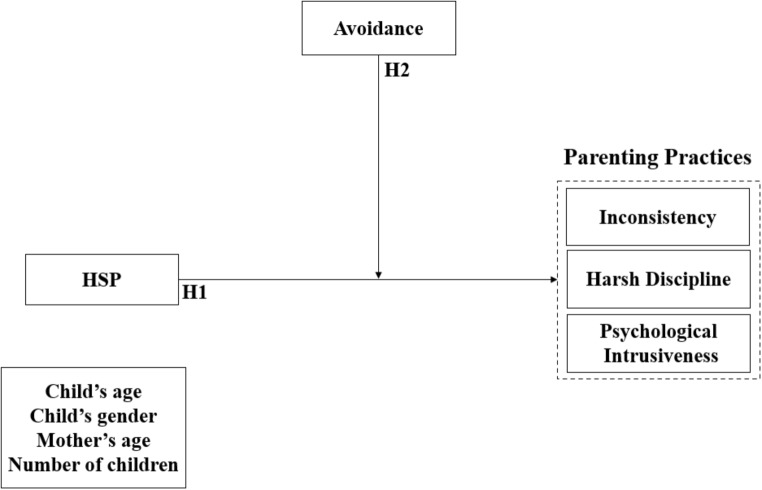
Simple slopes of HSP on inconsistency moderated by avoidance

The results from simple slopes analysis suggested that the slope for mothers with low avoidance was not significant. In other words, the analysis indicated that HSP did not have any significant effect on inconsistency for mothers with low avoidance (*β* = 0.02, n.s.). In contrast, for mothers with high avoidance, HSP was positively related to inconsistency (*β* = 0.30, *p* < .01) (see Fig. 2).

**Fig. 3:**
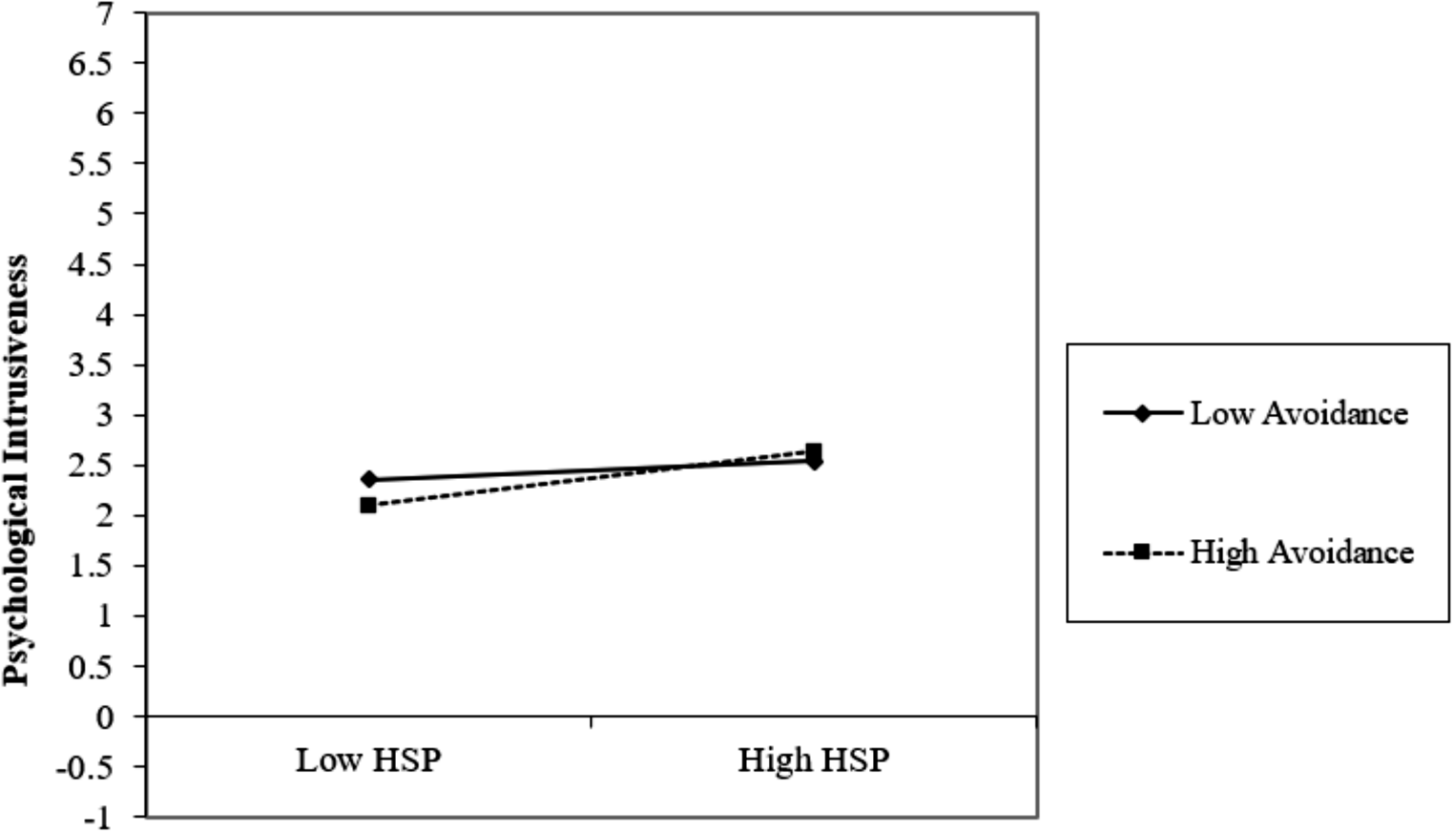
Simple slopes of HSP on psychological intrusiveness moderated by avoidance

We obtained similar results for effects of HSP on psychological intrusiveness moderated by avoidance. For mothers with low avoidance there was no significant effect of HSP on psychological intrusiveness (*β* = 0.10, n.s.). However, for mothers with high avoidance, HSP was positively related to psychological intrusiveness (*β* = 0.31, *p* < .01) (see Fig. 3).

## Discussion

There are well-established empirical studies that examined the emotional and social characteristics of highly sensitive individuals (e.g [22]). Yet there is very little research regarding HSPs parenting their children, especially during the challenging period of adolescence. Goldberg and Scharf [37] found that high sensitivity in parents was positively associated with inconsistency, psychological intrusiveness, and attachment anxiety and that attachment anxiety mediated the association between parents’ high sensitivity and harsh parenting and partially mediated the association between parents’ high sensitivity and parental psychological intrusiveness. We hypothesized that highly sensitive mothers also would exhibit negative parental practices with higher levels of inconsistency, psychological intrusiveness, and harsh discipline toward their adolescent children. Then we examined the contribution of attachment avoidance to the relationships between high sensitivity and negative parenting practices.

We found that the first hypothesis was mostly confirmed as highly sensitive mothers were reported by the adolescents to be inconsistent and intrusive. These results, compatible with prior research, showed that HSPs are prone to more negative emotional states under challenging conditions (e.g [21]). Hence, highly sensitive parents may experience parenting an adolescent child as especially challenging and more stressful. Their efforts to manage their own worries may decrease their ability to respond suitably to their adolescents’ needs. Moreover, acknowledging the burden of their sensitivity might affect their ability to cope with the parental challenges [27] and increase parenting difficulty [26], driving them toward a non-optimal parenting style [28] and practicing negative parenting behavior [29, 37].

The second hypothesis that avoidant attachment moderates the relationship between high sensitivity and negative parenting practices was also mostly confirmed, as the relationships between high sensitivity and inconsistent and intrusive parenting were strengthened with mothers` high level of attachment avoidance. This is an interesting finding regarding the role of attachment avoidance on highly sensitive persons since prior research had found no associations between attachment avoidance and HSP [18, 37]).

Attachment avoidance influences adult functioning such as information processing, emotion regulation, coping strategies, and parenting behavior [32, 38], more so for highly sensitive parents due to their being differentially susceptible to environmental factors [20].

Parental avoidant attachment orientation might drive the parent to misinterpret the adolescent’s needs as a threat, minimizing the parent’s involvement to avoid the distress caused by failed efforts to cope [40]. Hence, the avoidant parents, who use deactivation emotion regulation strategies believing that the best way to maintain safety is by not allowing the adolescent child to depend on them, demonstrate less caring and responsive parenting (e.g [54]). Moreover, HSPs are highly aware of others’ emotions as well as their own [55]. Thus, they may identify with the adolescent’s thoughts, emotions, and distress, which might, on the one hand, increase their own stress, while, on the other hand, their avoidant orientation and disapproving negative emotions might drive them to misinterpret their adolescent child. The experience of HSPs being differentially susceptible to environmental factors (e.g [20]). might be integrated into parenting behavior that might be experienced by the adolescent as inconsistent and intrusive.

Results indicated that parenting the adolescent is highly challenging for the highly sensitive mother and that it is associated with inconsistent and psychologically intrusive parenting. Furthermore, this is the first study that, in respect to HSPs being differentially susceptible to environmental factors [19, 23], investigates the moderating role of attachment avoidance between high sensitivity parenting and points out the vulnerabilities of highly sensitive mothers during the challenging period of adolescence. Hence, interventions focused specifically for avoidant highly sensitive mothers are needed. Such interventions might be especially impactful, as research shows that HSPs tend to benefit more from interventions [25].

## Limitations

Although the study contributes new information to the literature, there are some limitations. The current study employed a cross-sectional design. Hence, it is impossible to determine whether parenting practices were the result of the mothers’ high sensitivity or whether the challenged parenting increased the levels of high sensitivity. Moreover, the study used a convenience sample of mothers and adolescents and self-report questionnaires, and the reports might have been affected by other variables that were not tested. It is essential to bear in mind that adolescents’ evaluations of the parenting they experience also might be influenced by their own personalities, which were not assessed in the current research. Finally, the study was carried out within Israeli society and thus any conclusions regarding other societies need to be drawn cautiously. Future research should also address both parents, examine parents’ stress and other aspects of their personalities, examine adolescents’ adjustment to developmental tasks within other societies, and include other methods of assessment, such as interviews.

### Summary

Adolescence is a challenging period for children and parents due to major biological, cognitive, and emotional changes the adolescents undergo. This period is especially challenging within Israeli society, which is characterized by strong family values and connectedness, as well as high involvement of parents with their adolescent children’s lives, which enhances difficulties in parental monitoring and authority. In this context, the current study examined Israeli highly sensitive mothers’ parental practices toward their adolescent children and the role of attachment avoidance as a moderator between the associations of high sensitivity and parenting practices. Results showed that highly sensitive mothers practice inconsistent and intrusive parenting and that attachment avoidance moderates the association between mothers’ high sensitivity and inconsistent and psychological intrusiveness. Although there are some limitations to the current study, its findings add to the literature showing the differential susceptibility of HSPs to environmental factors, especially parenting tasks. Further investigation is needed to develop and improve interventions focused on the emotional regulation of HSP parents.

## Data Availability

No datasets were generated or analysed during the current study.
